# Supplementary material for: Impact of the WHO Safe Childbirth Checklist on safety culture among health workers: A randomized controlled trial in Aceh, Indonesia
Source: PLOS Glob Public Health. 2023 Jun 16;3(6):e0001801. doi: 10.1371/journal.pgph.0001801 (PMC10275423; doi:10.1371/journal.pgph.0001801)
Supplement: S1 Text — (DOCX) [file pgph.0001801.s001.docx]

**Supplementary Materials**

**Eligibility criteria**

Facilities were eligible if they provided at least basic emergency obstetric and newborn care services (BEmONC), which cover the provision of oxytocin and antibiotics, manual removal of the placenta, assisted vaginal delivery, abortion, and resuscitation of the newborn. Out of 40 eligible facilities, eight health facilities in those three districts did not participate in the study because they either did not report any births during the month before the baseline survey or asked for financial compensation, which we could not provide.

**Balance of health outcomes and facility characteristics**

Table A reports the balance of health outcomes and facility characteristics. The differences between treatment and control group are statistically insignificant, signaling balance at baseline. Within the control group we see a slightly lower proportion of hospitals (31%), higher proportion of facilities located in urban areas and fewer private clinics as compared to the treatment group. The public health insurance scheme BPJS covers 87% of delivery services in the treatment group, compared to 100% in the control group, which corresponds to the slightly higher proportion of private clinics in the control group. The accreditation level is measured as a categorical variable ranging from 1 to 5 indicating four accreditation levels (A provincial hospital, B district hospital, C and D hospitals) and a fifth for non-accredited primary care (puskesmas). Though insignificant, the control group has higher maternal sepsis and death rates, which could be linked to the higher share of primary care facilities in the control group, where existing referral structures and capacities may inhibit a timely referral to a secondary or tertiary care facility in case of complication during labor.

**Balance of individual attitudes**

We find significant differences for some measures (SpeakUp, ErrorExc, ResAcc) across staff at the baseline in Appendix Table B. Yet, one needs to take into account that the midwives for whom we collected those data at the baseline may differ from the ones at the endline due to staff turnover. Moreover, building on the qualitative feedback from the pre-treatment data collection, we adjusted selected questionnaire items to make them more applicable to the study context (e.g., InfoAccess1/2 were only collected post-treatment).

**Statistical analysis**

For treatment assignment, we used the publicly available [minMSE](https://sebseb.uber.space/minMSE/) code in Stata.^16^ We estimated Intention To Treat (ITT) effects and Complier Average Causal Effects (CACE) using Stata.

**Sample size**

Our research team collected data at 32 health facilities. However, due to changes in the accreditation system during the observation period, two facilities closed their delivery room. The team of interviewers thus interviewed 376 midwives across 30 health facilities. Aiming for a power of 80%, detectable effects are of magnitude of 0.9-1.1 of a standard deviation at the facility level (see Appendix Table C). Despite the high baseline levels of certain outcomes and a potential ceiling effect, distinct variation in the data makes the study sufficiently powered to estimate tangible effects.

**Minimal detectable effects**

Appendix Table C describes minimal detectable effect sizes for the attitudes. We estimate minimal detectable effects based on following formula:

$$MDE=\left( t_{1-\frac{\propto}{2}}+\rho\right)\sqrt{\frac{\sigma^{2}}{Np(1-p)}}$$

where MDE refers to the minimal detectable effect, $\boldsymbol{t}_{\boldsymbol{1-}\frac{\boldsymbol{\propto}}{\boldsymbol{2}}}$ to the level of intended statistical significance, $\rho$ the intended power (in our case 0.8), $\sigma^{\boldsymbol{2}}$ is the variance, N to the number of observations per cluster (1 as we consider the facility level) and p the fraction of observations in treatment and control group.

**Intention to Treat (ITT) analysis**

The basic estimation equation for the Intention to Treat (ITT) effect reads as follows:

$$\bar{Y_{i}} =\alpha+\beta_{1}T_{i}+{\beta_{2}X}_{i}+\varepsilon_{I}$$

where $Y_{i}$ refers to the safety culture measures averaged at the facility level, $T_{i}$ indicates if the facility was in the treatment group and $\varepsilon_{i}$ is the error term. $X_{i}$ is a vector of covariates (facility type, urban-rural, CeMonC-status, district dummy).

**Complier Average Causal Effect (CACE) analysis**

Our CACE approach builds on a two-step analysis, where the treatment allocation serves as an instrument to predict compliance in the first stage:

$$C_{ij}=\alpha+\beta_{1}T_{i}+\beta_{2}X_{i}+\varepsilon,$$

where $C_{ij}$ indicates average compliance (checklist use) at birthing process j at facility i. Predicted compliance $\hat{C}_{ij}$ is inserted into the second stage to estimate following model:

$$Y_{ij}=\alpha+\beta_{1}\hat{C}_{ij}+{\beta_{2}X}_{i}+\varepsilon_{I},$$

where $Y_{ij}$ refers to measures of health worker safety cultures at the facility level (health outcomes at facility j), which are regressed on predicted compliance. Compliance is measured at the facility level by calculating the number of completed SCCs over the total numbers of births. While facilities from the control group may also theoretically qualify as compliers if they would use the SCC, this case did not materialize during observed births [31]. Our clustered trial design (provision of SCC at facility-level) made spill-overs very unlikely to happen. Cragg-Donald Wald F statistics were larger than the critical rule of thumb value of 10, which suggests that the treatment is a sufficiently strong predictor of compliance to warrant reliable inference (e.g., we do not face weak instrumental variable issues which would otherwise inflate our estimates).

**Coaching approach**

The coaching is described in Appendix Table A7. Coaching comprises three visits in the first month, two visits in the second, third, and fourth month and finally, one visit in the fifth and sixth month. This coaching usually consisted of a two-hour visit of the coach at the facility and should comprise (i) a meeting with the checklist quality coordinator (CQC), (ii) filling out a short survey on checklist usage and barriers, and (iii) an opportunity for consultation on correct checklist use. Additionally, when possible, the coaches (iv) provided feedback on observed birthing processes and (v) gave input about the previously collected checklists. CQCs were selected among the midwives as a person with responsibilities to ensure regular use of the checklist and support other midwives with the application. The CQCs did not receive any remuneration.

**Checklist adaptation**

Together with Indonesian translators, we ensured a proper adaptation by forward and backward translation. Moreover, we cooperated with local health staff, to adjust the checklist to the local context and needs as listed below:

- Pause Point 2:
  - Confirm essential supplies are at bedside and prepare for delivery: Adapted item “Sterile blade to cut cord” to “Sterile scissors/knife to cut cord” to account for local practice
- Pause Point 3:
  - Is mother bleeding abnormally?: Added items “Yes, treat, but if cause cannot be treated, refer,” and “If cause cannot be treated, refer based on your criteria” to accommodate the fact that community health centers (puskesmas) may not be able to address abnormal bleeding
  - Start breastfeeding and skin-to-skin contact (IMD) (if mother and baby are well): Added item “No, start later because mother or baby are not well” to provide midwives with an option to check if patient’s status does not allow the starting of breastfeeding and skin to skin contact
- Pause Point 4:
  - Discuss and offer family planning options to mother: Add item “No, already done in antenatal care” and “No, will be done later” to allow midwives to check item if the point cannot be addressed immediately

Besides contextual adjustments, we added three major modifications according to practitioners’ feedback. First, we included a slot for SCC users to note the time and date of given medication. This documentation should make it easier for the next user to assess which medication was provided at what time and which further medication may be needed. Second, we included a field to add information on the mother to attach it to the patient file (e.g., mother’s name, age, weight, height). Third, we prepared a separate sheet for the danger signs to hand over to mothers and relatives before discharge, as a means for families to be better able to remember and assess situations in which they should return to the health facilities. We provide an English version of the adapted SCC in Fig A.

**Checklist use**

The paper-based checklist is attached to the patients file and travels with the patient from admission to discharge. Midwives were instructed by the coaches to fill-in the paper-based checklist at each pause point. Our team also provided a checklist poster for delivery rooms in order to allow midwives to follow the checklist during emergencies or when not working in teams (e.g., during nightshifts).

**Fig A: Adapted SCC**


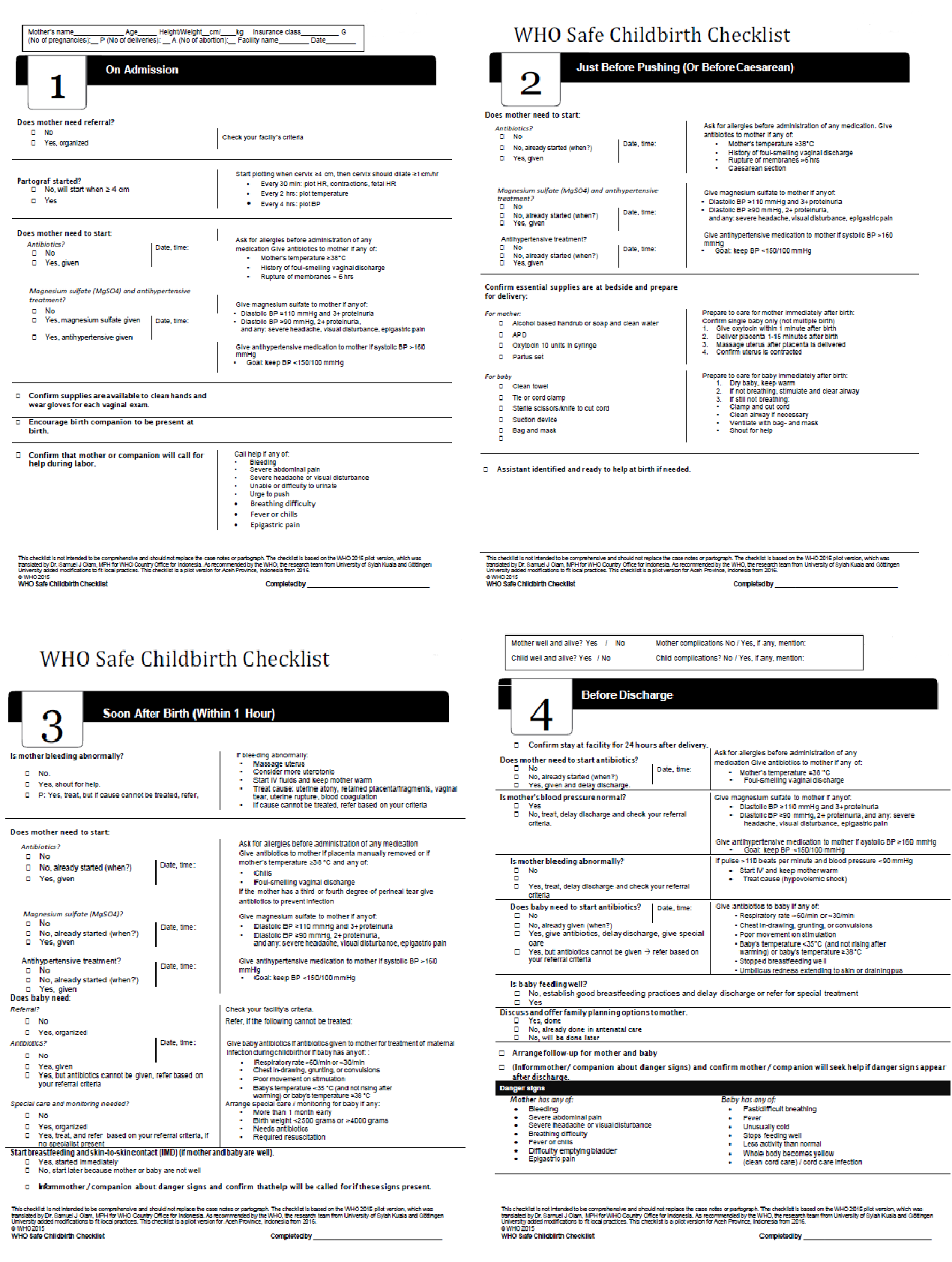


**Table A: Balance of health outcomes and facility characteristics**

|  | Control | Treatment | Difference | p-value | |
| --- | --- | --- | --- | --- | --- |
| *Facility characteristics* |  |  |  |  | |
| Hospital (1) & Primary Health Care (0)* | 0.33 | 0.47 | 0.13 | 0.47 | |
| Bireuen (1) & AcehBesar/Banda Aceh (0)* | 0.47 | 0.33 | -0.13 | 0.47 | |
| Urban (1) & Rural (0)* | 0.47 | 0.33 | -0.13 | 0.47 | |
| Public (1) & Private (0)* | 0.80 | 0.53 | -0.27 | 0.13 | |
| CeMonC 24 hours (1) & 1-23hours (0)* | 0.33 | 0.27 | -0.07 | 0.70 | |
| BPJS Delivery Coverage* | 1.00 | 0.87 | -0.13 | 0.15 | |
| Accreditation Level* | 4.27 | 4.14 | -0.12 | 0.77 | |
| Annual Deliveries** | 0.33 | 0.47 | 0.13 | 0.47 | |
| *Health outcomes* |  |  |  |  | |
| Maternal Death Rate*** | 189.46 | 83.08 | -106.38 | 0.4938 | |
| Still Birth Rate**** | 13.50 | 12.20 | -1.30 | 0.88 | |
| Newborn Death Rate**** | 2.66 | 2.12 | -0.53 | 0.81 | |
| Pre-Eclampsia Rate**** | 22.92 | 15.33 | -7.60 | 0.38 | |
| Eclampsia Rate**** | 7.03 | 3.89 | -3.15 | 0.57 | |
| Rupture of Uterus Rate**** | 0.08 | 0.14 | 0.06 | 0.72 | |
| Postpartum Hemorrhage Rate**** | 44.95 | 26.74 | -18.21 | 0.38 | |
| Rate of Infections among Mothers**** | 2.94 | 1.18 | -1.76 | 0.46 | |
| Obstructed and Prolonged Labor Rate**** | 50.92 | 87.37 | 36.45 | 0.42 | |
| Anesthetic Complications Rate**** | 2.04 | 2.12 | 0.08 | 0.98 | |
| Maternal Sepsis Rate**** | 45.17 | 2.19 | -42.98 | 0.17 | |
| Bad or foul-smelling vaginal discharge Rate**** | 0.41 | 3.69 | 3.29 | 0.33 | |
| Antepartum Hemorrhage Rate**** | 88.73 | 61.95 | -26.78 | 0.63 | |
| Birth trauma Rate**** | 17.63 | 9.11 | -8.51 | 0.65 | |
| Asphyxia Rate**** | 107.35 | 72.90 | -34.45 | 0.57 | |
| Hypothermia Rate**** | 2.26 | 0.78 | -1.48 | 0.53 | |
| Respiratory Distress Syndrome Rate**** | 10.03 | 4.53 | -5.50 | 0.51 | |
| Neonatal Sepsis Rate**** | 10.00 | 3.47 | -6.52 | 0.46 | |
| Prematurity Rate**** | 25.45 | 15.30 | -10.15 | 0.56 | |
| Short 4 Age Rate**** | 6.88 | 8.07 | 1.18 | 0.87 | |
| Low Birth Weight Rate**** | 54.34 | 39.14 | -15.20 | 0.55 | |
| Umbilical cord infection Rate**** | 3.54 | 1.18 | -2.36 | 0.42 | |
| Fever Rate**** | 2.95 | 1.42 | -1.53 | 0.53 | |
| Jaundice Rate**** | 4.00 | 4.34 | 0.34 | 0.91 | |
| N | 15 | 15 |  |  | |
| Notes: CEmONC services cover safe blood transfusion, provision of oxytocin and antibiotics, performance of cesarean sections, manual removal of the placenta, assisted vaginal delivery, removal of retained products, and resuscitation of the newborn.  Stars refer to * mean scores, ** frequencies, *** rate x/100,000, **** rate x/1000 | | | | |  |

**Table B: Balance of Individual Attitudes**

|  | Mean - Control | Mean - Treatment | ITT - No Controls | p-val |
| --- | --- | --- | --- | --- |
| Information Flow | 5.0994 | 5.0726 | -0.0268 | 0.7407 |
| Coordination | 5.1794 | 5.2204 | 0.0410 | 0.6762 |
| SpeakUp | 5.0194 | 5.1658 | 0.1464 | 0.0555 |
| FreqMissed | 1.5545 | 1.5620 | 0.0075 | 0.9568 |
| FreqUnsure | 1.4092 | 1.5920 | 0.1828 | 0.1644 |
| ErrorFatigue | 1.6131 | 1.5265 | -0.0866 | 0.5370 |
| ErrorExc | 1.8372 | 1.5215 | -0.3156 | 0.0834 |
| ErrorDist | 1.5067 | 1.3033 | -0.2034 | 0.3499 |
| ErrorKnow | 1.7843 | 1.5548 | -0.2295 | 0.3078 |
| Workload | 3.0249 | 2.8732 | -0.1517 | 0.1217 |
| Resource Access | 3.4184 | 3.6089 | 0.1904 | 0.0924 |
| Notes: Significance levels are indicated by stars refering to p-val *<10%, **<5%, ***<1%. Sample size:30 | | | | |

**Table C: Minimal detectable effect sizes for attitudes**

|  | Baseline Mean | ICC | Baseline SD | MDE (Alpha=0.1) | MDE (Alpha=0.05) |
| --- | --- | --- | --- | --- | --- |
| InformationFlow | 5.0860 | 0.5000 | 0.2166 | 0.1965 | 0.2214 |
| SpeakUp | 5.0926 | 0.5856 | 0.2108 | 0.1913 | 0.2155 |
| Coordination | 5.2000 | 0.0683 | 0.2619 | 0.2377 | 0.2678 |
| FreqMissed | 1.5582 | 0.2302 | 0.3690 | 0.3349 | 0.3773 |
| FreqUnsure | 1.5006 | 0.2800 | 0.3568 | 0.3238 | 0.3648 |
| ErrorFatigue | 1.5698 | 0.4582 | 0.3754 | 0.3406 | 0.3838 |
| ErrorExc | 1.6793 | 0.2263 | 1.5698 | 0.4533 | 0.5108 |
| ErrorDist | 1.4050 | 0.1058 | 0.5850 | 0.5308 | 0.5981 |
| ErrorKnow | 1.6696 | 0.1400 | 0.6058 | 0.5497 | 0.6194 |
| Workload | 2.9490 | 0.0450 | 0.2671 | 0.2424 | 0.2731 |
| Resource Access | 3.5134 | 0.2388 | 0.3097 | 0.2810 | 0.3166 |

SD: Standard deviation. ICC: Intra-cluster correlation at the facility level. MDE: Minimal detectable effect. Alpha=Confidence Interval Assumptions: Sample size: 30, power: 0.8.

**Table D: Robustness - Results with covariates**

|  | **Mean Control** | **Mean Intervention** | **Treatment**  **Effect** | **CACE** |
| --- | --- | --- | --- | --- |
| **Information Accessibility** |  |  |  |  |
| (1) **InfoAccess1:** During your shift, do you always have access to the following patient information: Diagnosis (Scale 1 [No access at all] to 4 [Full Access]) | 3.6689 | 3.6605 | 0.2422 [-0.619 - 1.104] | 0.5615 [-1.059 - 2.182] |
| (2) **InfoAccess2:** During your shift, do you always have access to the following patient information: Medication (Scale 1 [No access at all] to 4 [Full Access]) | 3.6271 | 3.670 | 0.4431 [-0.369 - 1.255] | 1.0272 [-0.540 - 2.594] |
| **Information Transmission** |  |  |  |  |
| (3) **InformationFlow:** Relevant information is communicated appropriately within the delivery team. (Scale 1 [Disagree strongly] to 6 [Agree strongly]) | 5.099 | 5.073 | 0.1817 [-0.591 - 0.955] | 0.4213 [-0.966 - 1.808] |
| (4) **SpeakUp:** In this clinical area, it is easy to speak up if I perceive a problem with patient care. (Scale 1 [Disagree strongly] to 6 [Agree strongly]) | 5.0194 | 5.1658 | 0.7944* [-0.045 - 1.633] | 1.8418** [0.239 - 3.445] |
| (5) **Coordination:** The delivery staff members here work together as a well-coordinated team. (Scale 1 [Disagree strongly] to 6 [Agree strongly]) | 5.1794 | 5.2204 | 0.2618 [-0.549 - 1.073] | 0.6069 [-0.840 - 2.054] |
| (6) **FreqMissed:** During your most recent delivery shift-week, how often did you forget to transmit important information during sign-out? (Scale 1 [Never] to 6 [Very often]) | 1.5545 | 1.5620 | -0.4312 [-1.274 - 0.412] | -0.9996 [-2.600 - 0.601] |
| (7) **FreqUnsure:** During your most recent delivery shift-week, how often did you report information that you were unsure of? (Scale 1 [Never] to 6 [Very often]) | 1.4092 | 1.5920 | 0.4815  [-0.331 - 1.294] | 1.1162 [-0.231 - 2.464] |

**Table D (ctd.): Robustness - Results with covariates**

| **Frequency of errors** |  |  |  |  |
| --- | --- | --- | --- | --- |
| (8) **ErrorKnow:** During your most recent delivery shift-week, how often did you make errors because of inadequate knowledge? (Scale 1 [Never] to 6 [Very often]) | 1.7843 | 1.5548 | -0.2473 [-1.145 - 0.650] | -0.5733 [-2.283 - 1.136] |
| (9) **ErrorFatigue:** During your most recent delivery shift-week, how often did you make errors because of fatigue? (Scale 1 [Never] to 6 [Very often]) | 1.6131 | 1.5265 | -0.2391 [-1.062 - 0.584] | -0.5543 [-2.068 - 0.959] |
| (10) **ErrorDist:** During your most recent delivery shift-week, how often did you make errors because of distractions? (Scale 1 [Never] to 6 [Very often]) | 1.5067 | 1.3033 | -0.3115 [-1.212 - 0.589] | -0.7221 [-2.446 - 1.002] |
| (11) **ErrorExc:** During your most recent delivery shift-week, how often did you make errors because of excessive workload? (Scale 1 [Never] to 6 [Very often]) | 1.8372 | 1.5215 | -0.5987 [-1.416 - 0.219] | -1.3880 [-3.124 - 0.348] |
| **Workload** |  |  |  |  |
| (12) **Paperwork:** Paperwork takes too much time. (Scale 1 [Disagree strongly] to 6 [Agree strongly]) | 2.8177 | 2.8887 | -0.0566 [-0.756 - 0.642] | -0.1313 [-1.415 - 1.153] |
| (13) **Workload:** How would you rate the average workload among your delivery staff at your health facility? (Scale 1 [Very low] to 5 [Very high]) | 3.0249 | 2.8732 | -0.2996 [-1.202 - 0.602] | -0.6945 [-2.410 - 1.021] |
| **Access to resources** |  |  |  |  |
| (14) **ResAcc:** Do you have access to the tools and resources to do your job well? (Scale 1 [Never] to 4 [Always]) | 3.4184 | 3.6089 | 0.3165 [-0.343 - 0.976] | 0.7339 [-0.560 - 2.028] |
| Observations | 15 | 15 | 30 | 30 |
| Notes: The dependent variable is depicted in standard deviations. 95% Confidence Intervals are depicted in brackets and significance levels are indicated by stars: p-val: *<10%, **<5%, ***<1%. F-Statistics for the CACE estimator is for all regressions {39.639}. Cragg-Donald Wald F statistics>10 suggest that the treatment is a sufficiently strong predictor of compliance to warrant reliable inference (e.g., we do not face weak instrumental variable issues). | | | | |

**Table E: Background information – Coaching approach**

| **Treatment** | **Control** |
| --- | --- |
| SCC exposure:   - 1x Checklist Introduction event (2 hours): Presentation, checklist explanation, role play (no training), selection of (non-remunerated) checklist quality coordinators - 11x Monitoring visits over six months (2 hours each): Checklist provision & collection, interviews with providers, feedback on previous performance, opportunity to ask questions - 2x Meetings of facility-based checklist quality coordinators (2 hours each) three and six months post introduction: Focus group discussions to exchange best practice - Checklist provision and provision of danger sign sheets   Exposure to the research team:   - Information event for facility leadership to present study design - Observations (24 hours over six days in larger facilities and one month on call in smaller facilities) - Survey on provider characteristics and attitudes (30-40 minutes) | SCC exposure:   - None   Exposure to the research team:   - Information event for facility leadership to present study design - Observations (24 hours over six days in larger facilities and one month on call in smaller facilities) - Survey on provider characteristics and attitudes (30-40 minutes) |

**Table F: Survey Items**

| **Information Accessibility** |
| --- |
| (1) **InfoAccess1:** During your shift, do you always have access to the following patient information: Diagnosis (Scale 1 [No access at all] to 4 [Full Access]) |
| (2) **InfoAccess2:** During your shift, do you always have access to the following patient information: Medication (Scale 1 [No access at all] to 4 [Full Access]) |
| **Information Transmission** |
| (3) **InformationFlow:** Relevant information is communicated appropriately within the delivery team. (Scale 1 [Disagree strongly] to 6 [Agree strongly]) |
| (4) **SpeakUp:** In this clinical area, it is easy to speak up if I perceive a problem with patient care. (Scale 1 [Disagree strongly] to 6 [Agree strongly]) |
| (5) **Coordination:** The delivery staff members here work together as a well-coordinated team. (Scale 1 [Disagree strongly] to 6 [Agree strongly]) |
| (6) **FreqMissed:** During your most recent delivery shift-week, how often did you forget to transmit important information during sign-out? (Scale 1 [Never] to 6 [Very often]) |
| (7) **FreqUnsure:** During your most recent delivery shift-week, how often did you report information that you were unsure of? (Scale 1 [Never] to 6 [Very often]) |
| **Frequency of errors** |
| (8) **ErrorKnow:** During your most recent delivery shift-week, how often did you make errors because of inadequate knowledge? (Scale 1 [Never] to 6 [Very often]) |
| (9) **ErrorFatigue:** During your most recent delivery shift-week, how often did you make errors because of fatigue? (Scale 1 [Never] to 6 [Very often]) |
| (10) **ErrorDist:** During your most recent delivery shift-week, how often did you make errors because of distractions? (Scale 1 [Never] to 6 [Very often]) |
| (11) **ErrorExc:** During your most recent delivery shift-week, how often did you make errors because of excessive workload? (Scale 1 [Never] to 6 [Very often]) |
| **Workload** |
| (12) **Paperwork:** Paperwork takes too much time. (Scale 1 [Disagree strongly] to 6 [Agree strongly]) |
| (13) **Workload:** How would you rate the average workload among your delivery staff at your health facility? (Scale 1 [Very low] to 5 [Very high]) |
| **Access to resources** |
| (14) **ResAcc:** Do you have access to the tools and resources to do your job well? (Scale 1 [Never] to 4 [Always]) |
