# Supplementary material for: Impact of the WHO Safe Childbirth Checklist on safety culture among health workers: A randomized controlled trial in Aceh, Indonesia
Source: PLOS Glob Public Health. 2023 Jun 16;3(6):e0001801. doi: 10.1371/journal.pgph.0001801 (PMC10275423; doi:10.1371/journal.pgph.0001801)
Supplement: Attachment — Submitted filename: Response.docx [file pgph.0001801.s002.docx]

Reviewer #1: This is an innovative study that measures the effects of using the WHO Safe Childbirth Checklist in the context of the safety culture of health services.
The article was analyzed according to CONSORT protocol criteria extension for cluster designs. The comments below indicate minor adjustments to be made to the text.
TITLE: It’s necessary to insert in the title the information that’s a cluster randomised trial study.
ABSTRACT:
Even knowing that there are word limits for the abstract, I strongly suggest that authors consider three aspects to be added:
1) the eligibility criteria of the clusters.
2) the number of clusters randomized to each group.
3) the primary outcomes (the four dimensions) as well as the information that primary outcome pertains to the cluster level.

**Response to reviewer:** *Thank you for pointing us to the missing information in the abstract. We added the missing details on the eligibility criteria and the size of the treatment and control group. We also included the primary outcomes and their correspondence to the cluster level.*

INTRODUCTION
At the end of the introduction, the authors report the results of the study itself. I suggest rewriting the text putting the hypotheses related to the use of the checklist more comprehensively, considering the dimensions of the safety culture analyzed, without reporting your own results that should be presented in another section.

**Response to reviewer:** *Thank you. We have removed the summary of the results from the introduction and stated the research question more clearly.*

METHODS
It’s unclear whether the number of subjects interviewed at baseline and after six months was the same, or whether there were losses.
Why in Appendix Tables A2 and A3 there are no results for the criteria Information Accessibility (InfoAccess1 and InfoaAccess2).
In Appendix Table 2 it’s not clear p value refer <10%, <5% or <1%.

**Response to reviewer:** *The main results presented build on a comparison of individual safety culture perceptions by 376 midwives post-intervention. Above those data, we collected also responses from 148 individual midwives before the intervention. While some of those respondents are also part of the data collected after the intervention, we do not include that information in our analysis. The reason is on the one hand that our post-intervention sample is more comprehensive, and on the other hand, we adapted our survey tools based on insights gained during the pre-intervention data collection. Given those survey updates, we did not collect results for the criteria InfoAccess1 and InfoAccess2 at baseline and cannot provide information on baseline balance and statistical power in Tables A2 and A3. Thank you for pointing us to this imprecision, which we now reflect in updates in section “Outcomes and data collection” as well as the “Balance of individual attitudes” (supplementary appendix).Thanks also for pointing us to the misleading notes under Table A2, which we now adapted to “Significance levels are indicated by stars refering to p-val *<10%, **<5%, ***<1%.”*

RESULTS:
It’s necessary to correct Figure 2. There is no correspondence with what is in table A4. For example, in Table A4 there are 14 safety culture criteria and in Figure there are 17. Furthermore, the text says that “the results show a significant increase in the probability that midwives would speak up when noticing patient care issues (row 4)”. However, in the figure, row 4 corresponds to "coordination”.
To understand the information in Figure 2, it is necessary to refer to Table A7 in the Appendix. For example: when analyzing the Figure, it is not clear what InfoAccess1 means. I suggest the authors describe the criteria that were analyzed in the text (on topic Outcomes and Data collection).

**Response to reviewer:** *We appreciate your comments. We addressed this mistake and adjusted Figure 2 to the results and correct order of Table A4. Now the text on the item SpeakUp also corresponds to the correct table row. Indeed, it was difficult to understand the variables in Figure 2 without referring to Table A7. We addressed this issue by including information on how we measured the four criteria in the section on Outcomes and data collection, as well as referring to the Appendix Table A7 (now Table A6). Additionally, we moved the table with the main outcomes in the text, where we outline the different variables and scales as well.*

**Reviewer #2: Review of manuscript PGPH-D-22-01265, entitled: “Impact of the WHO Safe Childbirth Checklist on Safety Culture among Health Workers: A Randomized Controlled Trial in Aceh, Indonesia”**

Thank you very much for this opportunity to review this manuscript. Studies on safety culture and safety checklists are interesting and important topics to study, as the authors have made clear. However, I do have some questions to the manuscript.
The study is described as a cluster randomized controlled trial. It is a bit unclear if it actually is a cross-over design in the trial? In a stepped wedge cluster RCT, the clusters serve as their own controls and the intervention is administered to all cluster, and at the end of the trial all clusters have received it. In a cluster RCT there will be ‘pockets’ of controls in the intervention period, which does not seem to be the case in this trial? At least it is difficult to assess whether this is the case or not. In the consort extention checklist, there is a reference to parallel or factorial trial design. It would be helpful if the design could be more clarified in the manuscript.
Did all 16 facilities receive the intervention at the same time point or at different time points? Again, this might have implications for the design used in the trial.
Who performed the randomization? Please add a description of this.
Randomisation – fasilities from the same health organization, mix or different organizations allocated into treatment or controls?

**Response to reviewer:** *We sampled for our study health facilities, which already offered at minimum basic emergency obstetric and newborn care (BEMonC). Those included community health clinics (puskesmas) as well as private and public hospitals. Via an optimization approach, we selected two equally sized groups of 16 facilities, in which observable characteristics (including facility/health organization type) were matched. Our study builds on a cluster-randomized trial, i.e., we introduced the checklist in 16 facilities and another 16 facilities served as a control group during the intervention period. During the intervention period, we do not provide any treatment (except the data collection) to the control group and, hence, did not rely on a cross-over or wedge-cluster design. The research team assigned treatment and control status randomly by coin toss and introduced the checklist to all treatment facilities in October 2016. Since coaches were responsible for multiple facilities, the introductory events were logistically spread over several days.*

*We included now additional details in the sections on “Intervention” and “Trial design/Randomization and masking” and hope to provide this way more clarity.*

Surveys were delivered on phone to participants. How was data privacy protection ensured for participants? How was data quality ensured? Please provide a brief description on this.
Paper checklist – how was it filled in/used? During the procedure, before or after? This could be described in more detail, to help the reader to understand the context of using the checklist.
Ethics. Do the authors have a reference number for ethical reviews from the ethical boards reviewing the study? Under ethics, it says that the study was reviewed, but not that it was approved. If it was approved prior to start, please mention this under this section.

**Response to reviewer:** *Thank you for those valuable suggestions. We addressed these very important issues by adding further detail in the sections on Outcomes and data collection, Intervention and Ethics.
The data was collected through face-to-face interviews on tablets and built on anonymized respondent IDs. At the end of the day, the data was uploaded by the enumerators to a secure server. No one but the researchers directly involved in the study had access to the data. After uploading, two researchers checked the data for consistency by enumerator. Feedback loops with enumerators took place after every data upload. Additionally, enumerators were accompanied on random occasions for spot checks.
The paper checklist was filled by midwives during the procedure and traveling with the patient from admission to discharge. In emergencies, midwives may have relied on a checklist poster, while filling-out the paper list ex-post.
The review boards approved/expressed no objection with respect to the final protocol prior to the trial. We added references to the declarations of the respective ethics boards in the text (“Universitas Syiah Kuala, Indonesia, (08/KE/FK/2016) and the ethics committee of the University of Göttingen, Germany (June 27, 2016)”).*

How many respondents filled in the survey? How many nurses/midwifes responded? What does 15 observations represent? Facilities or nurses? Please clarify this.
Compliance – who filled in the surveys? Did the survey respondents attend to the teaching classes?
Results – changes in safety culture – midwifes would speak up, is stated in Results, for both ITT and CACE. In Table A4 and A5, only CACE has a significant confidence interval. This means that the results are not accurately reported, in results or in the tables. This need to be corrected.

**Response to reviewer:** *Thank you for pointing us to this imprecision. We addressed this by a more elaborate description in the section on Outcomes and data collection. In total, 376 midwives were interviewed in the endline data collection. As the intervention was introduced at facility level, we estimate outcomes at the facility level, for which the 15 observations represent the 15 facilities each in treatment and control group. The individual questionnaires were filled by trained enumerators in computer assisted personal interviews with midwives. However, compliance is measured as a continuous indicator based on the number of completed checklists over the total number of births, which were collected on a regular basis by the coaches. Based on our treatment-control design, the midwives from the 15 treatment facilities participated in the teaching component of the study, whereas the midwives from the 15 control facilities received no teaching. Thank you also for pointing us to the mismatch between Tables A4 and A5 as well as the main results in Figure 2. We carefully reviewed the results and updated Figure 2, which now corresponds to the estimated tables in the appendix.*

Table A1 In the columns, what are the numbers (except * and **) representing? Mean scores or frequencies? This may be difficult to assess for the readers. Please clarify what all numbers represent. What are the scores for each variable in Table A1?
In Table A3 ,ICC is described under the table but it is not listed in the columns? What is the ICC values here? This needs to be more detailed.
The study include four dimensions, which the authors align with a safety culture. These are named: “information transmission”, “information accessibility”, “frequency of errors”, and “workload”. There is no data on the reliability of these dimensions. Please provide this for all four.
2

**Response to reviewer:** *Thank you for pointing us to this unclarity. We added notes for the respective mean scores and frequencies in Table A1, as well as adding a short description of some outcomes in the table in the appendix (see under section Balance of health outcomes and facility characteristics). We hope this sufficiently clarifies the interpretation of the values in Table A1.*

*Although we aggregate in the main analysis outcomes per cluster (Facility), we report now in Table A3 also the intra-cluster correlation.*

*We have also rewritten the conclusions and interpretation of results such that we provide evidence that the SCC can affect some dimensions of safety culture while we find no evidence for effects in other dimensions.*

**Reviewer #3: Thank you for the opportunity to review this informative manuscript. There is a clear and compelling need to explore ways that may lead to culturally appropriate implementation of the Safe childbirth Checklist (SCC). The issue of perceptions toward safety practices among providers of facility delivery care is important and not well explored previously. Please see detailed comments in the attached file.**

Impact of the WHO Safe Childbirth Checklist on Safety Culture among Health Workers:

A Randomized Controlled Trial in Aceh, Indonesia

PGPH-D-22-01265

Thank you for the opportunity to review this informative manuscript. There is a clear and compelling need to explore ways that may lead to culturally appropriate implementation of the Safe childbirth Checklist (SCC). The issue of perceptions toward safety practices among providers of facility delivery care is important and not well explored previously.

The paper does a good job delineating the need for such research. Data analysis is extensive and provides rich information about the perceptions and attitudes of nurse-midwives toward a safety culture. There are several aspects of the manuscript that could be improved.

**Abstract**

It lacks the research question. The main findings would benefit from including the results of the regressions

**Response to reviewer:** *Thank you. We have added this information to the abstract.*

**Introduction**

The authors identify other literature on the topic of SCC introduced by WHO in 2015 as a patient safety tool to improve facility-based births.

There is increasing evidence that the SCC is a vital tool in reducing perinatal mortality when implemented as part of broader quality improvement initiative.

Varghese B, Copas A, Kumari S, et al. Does the safe childbirth checklist (SCC) program save newborn lives? Evidence from a realistic quasi-experimental study, Rajasthan, India. *Matern Health Neonatol Perinatol*. 2019;5(3). [doi:10.1186/s40748-019-0098-4](https://doi.org/10.1186/s40748-019-0098-4)

Walker D, Otieno P, Butrick E, et al. Effect of a quality improvement package for intrapartum and immediate newborn care on fresh stillbirth and neonatal mortality among preterm and low-birthweight babies in Kenya and Uganda: a cluster-randomised facility-based trial. *Lancet Glob Health*. 2020;8(8):e1061-e1070. [doi:10.1016/s2214-109x(20)30232-1](https://doi.org/10.1016/s2214-109x(20)30232-1)

The authors claim that there is little evidence from non-randomized studies on how the SCC may affect the safety culture. I suggest looking closer. A recent study in Brazil indicated that the context-specific factors, such as facility infrastructure, supply chains, teamwork culture, and quality improvement capacity, and adherence to the SCC seem to modulate its impact on birth outcomes.

Sousa KM, Saturno-Hernández PJ, Rosendo TMSS, et al. Impact of the implementation of the WHO Safe Childbirth Checklist on essential birth practices and adverse events in two Brazilian hospitals: a before and after study. BMJ Open. 2022;12(3):e056908. Published 2022 Mar 14. doi:10.1136/bmjopen-2021-056908

While the objectives of the study are stipulated in the introduction (impact of the SCC on the safety culture and investigate individual adherence to the SCC), they are not very well defined. It would be helpful to include in the introduction the safety culture dimensions investigated (i.e., the “four dimensions of safety culture through which the adoption of the checklist could support adherence to essential practices during childbirth).” It is also worth mentioning that the constructs measured were perceptions for these dimensions at the cluster level, so not exactly a direct study of safety culture at the providers’ level. Similarly, any literature review on compliance adherence among providers and approaches used to measure it would help place the manuscript into context.

**Response to reviewer:** *Thank you for your thorough reading of our study and the thoughtful comments. We particularly thank you for pointing us to the paper published by de Meneses Sousa and others, which adds to the motivation of considering safety culture dimensions in health interventions. We included this paper, as well as the Walker et al. (2020) in the introduction. The suggested paper by Varghese and others is considered in the systematic review of Tolu et al. (2020), for which we have not included it in the introduction, but cite it in the discussion. In addition, we added a brief overview on compliance measures in previous SCC studies in the introduction to provide context for the manuscript. We have further defined the dimensions of safety culture more clearly in the introduction and also mention that it was measured at the cluster level.*

**Methods:**

A brief summary of the protocol is needed. Specifically, more details are warranted about how the facilities were identified as eligible, it is unclear the mix of tertiary with lower level facilities, how the facility volume was taken into account (they should have been selected with a minimum of births per month and we find out that 2 facilities were dropped because did not have births for 6 months), how was basic emergency obstetric care assessed, were all providers midwives or nurses too, how was the sample size calculated.

There is also a repetition of Figure 1 and then another figure, unlabeled, that show that in fact 41 facilities were eligible and 9 not 8 refused to participate.

**Response to reviewer:** *We updated the Methods section and provide further detail on the sampling strategy under the heading “Trial setting and participants.“ More specifically, we clearly state in the updated manuscript that we included facilities that offered at least basic emergency obstetric and newborn care services (BEMonC). Based on this approach, 40 facilities were eligible. Out of those eight facilities refused to participate and a further two closed down their delivery rooms due to a change in the accreditation scheme during the survey period. This leaves us with a sample of 30 health facilities, which we now reflect clearer in the main text and appendix. For this purpose, we made also sure to include the correct figure on the sampling design. Moreover, we now also indicate in this section that we interviewed only midwives and that the sample size was based on power calculations for clinical outcomes in an accompanying paper (Kaplan et al., 2021), but we ensured sufficient power by checking the minimal detectable effects on safety culture measures in Table A2.*

**Statistical analysis**

Include the equation of the OLS regressions. As one of the tables (A5) includes covariates, please explain their selection. This may be a randomized study design, but the facilities may have been dissimilar enough to warrant the addition of covariates in the regressions.

**Response to reviewer:** *We added the OLS regression equation to the main text and state that we use the covariates to increase statistical efficiency. Moreover, we refer now also more explicitly to the equation in the supplementary appendix for the Complier Average Causal Effect. We hope that these changes increase clarity on our empirical approach.*

**Results**

The Tables illustrating the results need to be part of the manuscript not the appendix.

The treatment facilities have a much higher maternal mortality and sepsis rate. This may indicate that quality control measures may be different between the treatment and control groups. Perhaps a brief explanation of these differences is warranted.

Small detail, please do not use “abortion” in defining the emergency obstetric care (EmOC) life-saving interventions; replace with “removal of retained products” as EmOC is originally defined.

**Response to reviewer:** *We moved the table describing the main outcomes (previously Table A4, now Table 1 “Health Worker Safety Culture”) to the main text to improve readability. We replaced “abortion” with “removal of retained products”. Thank you for pointing us to this mistake. Concerning the higher maternal mortality and sepsis rate, the differences between control and treatment group are indeed higher, yet, insignificant. A potential explanation for this may lie the higher share of primary care facilities in the treatment group, where the existing referral structures and capacities in case of complications may inhibit a timely referral to a tertiary care facility. We added a paragraph describing the balance outcomes and this difference in maternal mortality and sepsis rate in Table A1 in the appendix under the section “Balance of health outcomes and facility characteristics”.*

**Discussion**

The authors are comparing the study results with other studies that focused on quality of care and mortality reduction, which is valuable. However, there are other examples in the literature about SCC and mortality reduction. More importantly, this research focuses on providers perceptions to sustained SCC use and comparison with other studies warrant more detail.

The only significant difference (at P<0.95) is in providers easiness “to speak up if I perceive a problem with patient care.” The lack of difference between the treatment and control group in other outcomes would need more discussion.

The authors did not include limitations of the study. Suggest adding.

**Response to reviewer:** *We added further literature to the sections Introduction and Discussion, which allows for a comparison of effects on both mortality and perceptions (e.g., Walker et al., 2019; Varghese et al., 2019; de Barros et al., 2021; de Moraes Custódio et al., 2021). The mixed results on mortality – some studies find an effect, others not – and qualitative evidence on health workers’ resistance towards the checklist help us to stress the relevance of researching safety culture. We now discuss more carefully the dimensions of safety culture that remain unaffected by the intervention. And we have added limitations in the discussion section.*
